# Psychiatric Emergency Presentations in Detained Adolescents: A Comparison With Community‐Living Adolescents

**DOI:** 10.1002/cbm.70051

**Published:** 2026-09-16

**Authors:** Maria Carolina Lobato Machado, Marcela de Moura Rezende, Izabela Lara Borém, Maria Eugênia Rezeck Braga Hibner, Danilo Barcellos Nogueira, Luciana Rodrigues da Cunha, Rodrigo Nicolato, Debora Marques de Miranda

**Affiliations:** ^1^ Postgraduate Program in Molecular Medicine Faculty of Medicine Federal University of Minas Gerais Belo Horizonte Brazil; ^2^ Faculdade de Ciências Médicas de Minas Gerais Belo Horizonte Brazil; ^3^ Fundação Hospitalar do Estado de Minas Gerais Belo Horizonte Brazil

**Keywords:** adjustment disorder, adolescent, conduct disorder, juvenile delinquency, juvenile justice, psychiatric emergency service, stress disorders, substance‐related disorders, suicide, traumatic

## Abstract

**Background:**

Adolescents involved in the juvenile justice system have high rates of psychiatric disorder and social vulnerability. Those in custodial or semi‐custodial detention may be particularly vulnerable to psychiatric emergencies, but the clinical factors associated with these crises remain insufficiently understood.

**Objective:**

To characterise the sociodemographic and clinical profiles of detained adolescents attending a psychiatric emergency service and compare them with those of community‐living adolescents.

**Methods:**

In this retrospective cross‐sectional study, we reviewed the medical records of all patients presenting to a public child and adolescent psychiatry emergency service in Brazil over 1 year. Detained adolescents aged 12–17 years were compared with community‐living adolescents regarding sociodemographic characteristics, psychiatric diagnoses and presenting complaints.

**Results:**

Of 1206 adolescents, 160 (13%) were detained. Compared with community‐living adolescents, detained adolescents were more likely to be male (86%: 53%), older (mean [SD] 15.9 [1.1]: 14.9 [1.6] years) and to identify as Black (13%: 8%). Stress‐related/adjustment disorders (30%:10%), conduct disorder (27%:10%) and substance use disorders (26%:20%), were more frequent among detained adolescents, whereas depression was less frequent (26%:35%). Emergency consultations involving detained adolescents were more likely to include insomnia (43%: 19%) and suicide attempts (26%: 18%), whereas behavioural presentations such as agitation, aggressiveness, irritability, disobedience and attention difficulties were comparatively less frequent.

**Conclusion:**

Emergency psychiatric presentations among detained adolescents were more strongly associated with stress‐related disorders, suicidal behaviour, sleep disturbance and substance‐related problems than with behavioural disturbance alone. These findings have implications for emergency psychiatric assessment within juvenile justice settings.

## 1 Introduction

Since 1990, Brazilian legislation has provided a legal framework for the protection of children and adolescents, granting them specific legal rights. Within this framework, children younger than 12 years cannot be held legally responsible for unlawful acts. Adolescents aged 12–17 years are also exempt from adult criminal prosecution; instead, unlawful conduct is classified as an ‘infraction’ rather than a crime and is addressed through the juvenile justice system (Brasil, Conselho Nacional de Justiça 2012; Brasil 1990). Infractions in Brazil are dealt with through socio‐educational measures, which integrate accountability with rehabilitative principles. These measures range from community‐based supervision to custodial detention, including semi‐custodial residential placement and custodial detention in state‐run facilities (Costa and da Silva 2017). Although the terminology differs from that used in many other jurisdictions, these socio‐educational measures are functionally comparable to sanctions used in international juvenile justice systems, such as probation, residential placement and juvenile detention.

Justice involvement in adolescence is commonly associated with social vulnerability and mental disorders (Gama et al. 2014). An updated systematic review and meta‐regression analysis reported that a substantial proportion of adolescents in juvenile detention and correctional facilities meet criteria for at least one mental disorder (Beaudry et al. 2021). Individual studies report prevalence ranging from 44% to 90%, depending on methodology (Bronsard et al. 2025; Teplin et al. 2021; Lim et al. 2018; Colins et al. 2010). Specifically, such young people have higher rates of externalising, mood, trauma‐related, and substance‐related disorders than their community‐living peers (Bronsard et al. 2025; Beaudry et al. 2021; Teplin et al. 2021; Lim et al. 2018; Colins et al. 2010; Abram et al. 2003), and these conditions often persist after release (Teplin et al. 2021; Abram et al. 2015). In Brazil, up to 80% of justice‐involved young people have at least one mental disorder (Dória 2011).

This situation is made worse by systemic barriers to treatment, including limited availability of child and adolescent psychiatry specialists and inadequate infrastructure within juvenile facilities (Resende and Volpe 2021; Porter et al. 2016). Consequently, mental health care is often reactive, occurring during acute crises in emergency settings (Lo et al. 2020; Mutlu et al. 2015). Although mental disorder prevalence among justice‐involved adolescents is well‐documented, the clinical drivers that precipitate psychiatric emergency presentations remain underexplored. Identifying these acute clinical presentations is crucial for informing preventive strategies and continuity of care. Therefore, this study aimed to characterise the sociodemographic and clinical profiles of detained adolescents attending a psychiatric emergency service and compare them with those of community‐living adolescents.

## 2 Methods

### 2.1 Ethical Statement

Ethical principles for scientific research were followed. Date of birth and medical record number were used solely for record linkage before anonymisation to protect participant identity. Data collection was reviewed and approved by the Ethics and Research Committee (CAAE: 83419717.0.0000.5134).

This study involved no direct participant contact, using only pre‐existing medical records. Consequently, the institutional ethics committee waived the requirement for informed consent.

### 2.2 Study Design

This cross‐sectional and retrospective study analysed data from medical records of all patients who presented over a 1‐year period (1 June 2017–31 May 2018) at the emergency department of a 24‐h public child and adolescent psychiatric service in Belo Horizonte, Brazil. As a regional facility, in a state population of 20 million, the service followed an attendance protocol. Initial clinical assessment was conducted by a multidisciplinary non‐medical mental health team. Following team review, cases requiring psychiatric intervention were then referred to an on‐call psychiatrist, whereas patients presenting with severe agitation or overt aggressiveness received immediate psychiatric evaluation.

### 2.3 Sample

Medical records of all patients younger than 18 years presenting during the study period were reviewed. The only exclusion criterion was age 18 years or older at presentation.

As custodial detention under the Brazilian juvenile justice system applies exclusively to adolescents aged 12–17 years (Brasil 1990), analyses were restricted to this age group. The detained group comprised adolescents in custodial or semi‐custodial detention. The comparison group comprised all other community‐living adolescents attending the same psychiatric emergency service during the study period. The sample was defined by each adolescent’s first presentation in the period, to avoid double‐counting, but any subsequent visits were documented.

### 2.4 Data Collection and Variables

Data were extracted using a standardized form by trained researchers under the supervision of a board‐certified child and adolescent psychiatrist. Although formal inter‐rater reliability was not assessed, regular supervisory meetings ensured extraction consistency. Variables included sociodemographic data (age, sex, self‐declared ethnicity, residence, education and legal guardianship) and clinical history (presenting complaints, prior diagnoses, comorbidities, trauma and family psychiatric history).

Detention status was verified through custodial documentation at presentation, including legal guardianship by state representatives. Given their legal status, these adolescents were escorted to the emergency department by socio‐educational staff, who acted as temporary legal guardians during the clinical evaluation.

Because of the acute nature of emergency care, standardized screening tools were not used; instead, diagnoses followed the International Statistical Classification of Diseases and Related Health Problems 10th Revision (ICD‐10) criteria established during psychiatric evaluation. Consultations performed solely by non‐medical staff were included for descriptive purposes, but diagnostic classification was restricted to physician‐documented cases to ensure clinical rigour.

Presenting complaints were derived from routine documentation and coded as categorical indicators: presence (explicit positive record), negative (explicit negative record) or missing (no documentation). Substance‐related diagnoses followed ICD‐10 criteria. Notably, among detained adolescents, these records were more likely to reflect prior substance use or abstinence‐related symptoms rather than acute intoxication, as access to illicit substances is legally prohibited within custodial settings. All eligible patients were included, and to address variability in routine records, analyses used available‐case denominators for each variable. Data were anonymised prior to analysis.

### 2.5 Statistical Analysis

Descriptive analysis included frequencies and percentages for categorical variables, which were analysed using Pearson’s Chi‐squared test or Fisher’s exact test, as appropriate. For continuous variables, means and standard deviations were calculated, and comparisons were performed using the independent samples Student’s *t*‐test. Statistical analysis was conducted using the Statistical Package for Social Sciences (SPSS [IBM, United States]) version 20.0. The significance level was set at 5%.

## 3 Results

### 3.1 Characterisation of the General Sample

Among the 1805 patients who attended the service, 127 (10.5%) required intensive treatment, such as inpatient care or a day hospital.

Of the total sample, 1206 were adolescents aged 12–17 years (mean age 15.0 ± 1.6 years; 689, 57% male). Among the 1126 adolescents with information on self‐declared ethnicity, nearly two‐thirds identified as brown (759, 67%), just over one‐fifth as white (266, 24%) and 99 (9%) as black. Most of these young people (662, 55%) resided in the capital city of Belo Horizonte, whereas 357 (30%) lived in municipalities elsewhere in the state and 187 (15%) in municipalities within the Belo Horizonte Metropolitan Region. Among those with information available, 735 (76%) were attending school. Information on the history of school grade repetition was available for 343 adolescents, of whom 161 (47%) had repeated at least one school year.

### 3.2 The Detained Adolescents

During the study period, there were 1738 emergency consultations by 1206 adolescents 12–17 year. The sample was defined by the first presentation in the period, to avoid double counting, but any subsequent visits were documented, indicating repeated visits by some of them. The following analyses compare 160 detained adolescents with the 1046 community‐living adolescents.

#### 3.2.1 Sociodemographic Characteristics

The detained adolescents were just over five times as likely to be male than in the comparison group, and they were significantly older (see Table 1). Among those with ethnicity data (1126/1206), detained adolescents were significantly less likely to identify as white and significantly more likely to identify as black, but there were no other significant differences.

**TABLE 1:** Comparison of the sociodemographic and clinical characteristics of detained and community‐living adolescents.

Variable	Detained adolescents, *n* (%)	Community‐living adolescents, *n* (%)	OR	95% CI	*p*
Sociodemographic characteristics					
Age					
Mean ± standard deviation	15.9 ± 1.1	14.9 ± 1.6	—	—	< 0.001
Sex					
Male	137 (85.6)	552 (52.8)	5.31	3.37–8.43	< 0.001
Female	23 (14.4)	494 (47.2)			
Attending school^a^					
Yes	48 (52.2)	687 (78.8)	0.29	0.19–0.46	< 0.001
No	44 (47.8)	185 (21.2)			
Repeated at least one school year^b^					
Yes	18 (85.7)	143 (44.4)	7.51	2.17–26.00	< 0.001
No	3 (14.3)	179 (55.6)			
Self‐declared ethnicity^c^					
White	23 (16.1)	243 (24.7)	0.58	0.36–0.93	0.02
Brown	100 (69.9)	659 (67.0)	1.14	0.78–1.67	0.49
Black	19 (13.3)	80 (8.1)	1.73	1.01–2.95	0.04
Yellow	1 (0.7)	1 (0.1)	6.91	0.43–111.18	0.11
Family history^d^					
Any psychiatric disorders	37 (56.1)	458 (76.7)	0.39	0.23–0.65	< 0.001
Substance use disorders^g^	22 (66.7)	151 (36.7)	3.46	1.63–7.33	0.001
Previous psychiatric history^e^					
Any psychiatric diagnoses	42 (34.4)	454 (52.7)	0.47	0.32–0.70	< 0.001
Substance‐related diagnosis^g^	15 (9.4)	19 (1.8)	5.6	2.78–11.25	< 0.001
Trauma history^f^					
Yes	159 (99.4)	337 (70.5)	66.5	9.22–479.86	< 0.001
No	1 (0.6)	141 (29.5)			

*Note:* Data are presented per individual patient.

Abbreviations: 95% CI = 95% confidence interval, OR = odds ratio.

^a^ data from 964/1206 patients.

^b^ data from 343/1206 patients.

^c^ data from 1126/1206 patients.

^d^ data from 663/1206 patients.

^e^ data from 983/1206 patients.

^f^ data from 638/1206 patients.

^g^ Substance use disorders are included within the category of any psychiatric disorder/diagnosis and are presented separately because of their clinical relevance.

School attendance was significantly lower among detained adolescents under socio‐educational measures, although there were substantial missing data for this variable (see also Table 1). Most striking, adolescents in custodial or semi‐custodial detention were seven times more likely to have repeated at least one school year, however, information on this variable was available for only about one quarter of the study sample.

#### 3.2.2 Family History, Past Psychiatric History and Trauma History

Family history, previous psychiatric history and trauma history are summarised in Table 1. Compared with community‐living adolescents, detained adolescents were less likely to have any documented family psychiatric history or any previous psychiatric diagnosis. However, within these broader categories, they were substantially more likely to have family and personal histories of substance use disorders. Trauma history was highly prevalent in both groups and was nearly universal among detained adolescents.

#### 3.2.3 Consultation Characteristics

Consultation characteristics are summarised in Table 2. Consultations involving detained adolescents were less likely to occur during daytime hours than those involving community‐living adolescents. In addition, detained adolescents were more likely to undergo psychiatric evaluation following the initial multidisciplinary assessment.

**TABLE 2:** Comparison of consultation characteristics between detained and community‐living adolescents.

Variable	Detained adolescents, *n* (%)	Community‐living adolescents, *n* (%)	OR	95% CI	*p*
Time of consultation					
Daytime consultation	172 (63.5)	1149 (78.3)	0.48	0.36–0.63	< 0.001
Night‐time consultation	99 (36.5)	318 (21.7)			
Psychiatric evaluation performed					
Yes	247 (91.1)	1214 (82.8)	2.14	1.38–3.33	0.001
No	24 (8.9)	253 (17.2)			

*Note:* Data are presented per consultation.

Abbreviations: 95% CI = 95% confidence interval, OR = odds ratio.

#### 3.2.4 Psychiatric Diagnoses Recorded During Emergency Consultations

Diagnostic analyses were performed at the consultation level because individual adolescents could attend the emergency service more than once and receive different psychiatric diagnoses across visits. Therefore, all emergency consultations were included in this analysis rather than only the first consultation. Diagnostic comparisons are summarised in Table 3.

**TABLE 3:** Psychiatric diagnoses recorded during emergency consultations involving detained and community‐living adolescents.

Variable	Detained adolescents, *n* (%)	Community‐living adolescents, *n* (%)	OR	95% CI	*p*
Depression	63 (25.8)	415 (35.3)	0.64	0.47–0.87	0.004
Bipolar disorder	10 (4.1)	59 (5.0)	0.81	0.41–1.60	0.537
Unspecified mood disorder	26 (10.7)	69 (5.9)	1.91	1.19–3.06	0.007
Unspecified psychosis	18 (7.4)	103 (8.8)	0.83	0.49–1.39	0.47
First psychotic episode	13 (5.3)	94 (8.0)	0.64	0.35–1.17	0.14
Schizophrenia	7 (2.9)	54 (4.6)	0.61	0.27–1.36	0.22
Anxiety disorder	5 (2.0)	50 (4.3)	0.47	0.18–1.19	0.10
Stress‐related/adjustment disorders	75 (30.4)	111 (9.5)	4.17	2.98–5.82	< 0.001
Dissociative conversion disorder	6 (2.5)	38 (3.2)	0.75	0.31–1.80	0.52
Obsessive‐ compulsive disorder	1 (0.4)	13 (1.1)	0.37	0.05–2.81	0.31
Attention‐deficit hyperactivity disorder	5 (2.0)	42 (3.6)	0.56	0.22–1.44	0.22
Oppositional defiant disorder	3 (1.2)	28 (2.4)	0.51	0.15–1.68	0.26
Conduct disorder	66 (27.0)	120 (10.2)	3.25	2.31–4.57	< 0.001
Mixed disorders of conduct and emotions	12 (4.9)	71 (6.1)	0.80	0.43–1.50	0.48
Substance use disorder	63 (25.6)	234 (20.0)	1.38	1.00–1.90	0.048
Intellectual disability	31 (12.7)	167 (14.2)	0.88	0.58–1.32	0.52
Autistic spectrum disorder	6 (2.5)	46 (3.9)	0.61	0.26–1.46	0.27
Learning disorder	0 (0.0)	3 (0.3)	1.21	1.18–1.24	0.42

*Note:* Data are presented per consultation.

Abbreviations: 95% CI = 95% confidence interval, OR = odds ratio.

Compared with community‐living adolescents, detained adolescents were significantly less likely to receive a diagnosis of depression, but more likely to receive a diagnosis of unspecified mood disorders, stress‐related/adjustment disorders, conduct disorder and substance use disorder. Other disorders, including psychotic disorders and neurodevelopmental conditions, were similar between the groups.

#### 3.2.5 Presenting Complaints Recorded During Emergency Consultations

Multiple presenting complaints could be recorded for each emergency consultation; therefore, complaint categories were not mutually exclusive. Among consultations involving detained adolescents, 19 (7%) had one presenting complaint, 35 (13%) had two presenting complaints and 216 (80%) had three or more.

The most frequent presenting complaints recorded during emergency consultations involving detained adolescents were prior substance use (44%), insomnia (43%), sadness (30%), agitation (29%), aggressiveness (27%) and suicide attempts (26%) (see Table 4 for details).

**TABLE 4:** Comparison of presenting complaints recorded during emergency consultations involving detained and community‐living adolescents.

Variable	Detained adolescents, *n* (%)	Community‐living adolescents, *n* (%)	OR	95% CI	*p*
Suicide attempts	71 (26.2)	267 (18.2)	1.60	1.18–2.16	0.002
Suicidal ideation	56 (20.7)	287 (19.6)	1.07	0.78–1.48	0.676
Self‐mutilation	58 (21.4)	312 (21.3)	1.01	0.73–1.38	0.960
Sadness	82 (30.3)	521 (35.5)	0.79	0.59–1.04	0.095
Feel anxiety	65 (24.0)	311 (21.2)	1.17	0.86–1.59	0.306
Agitation	79 (29.2)	529 (36.1)	0.73	0.55–0.96	0.028
Aggressiveness	72 (26.6)	557 (38.0)	0.59	0.44–0.79	< 0.001
Hallucinations	62 (22.9)	267 (18.2)	1.33	0.97–1.82	0.071
Delusions	28 (10.3)	172 (11.7)	0.87	0.57–1.32	0.509
Academic underachievement	15 (5.5)	279 (19.0)	0.25	0.15–0.43	< 0.001
Attention deficits	9 (3.3)	145 (9.9)	0.31	0.16–0.62	< 0.001
Angry behaviour/irritability	70 (25.8)	468 (31.9)	0.74	0.55–0.99	0.047
Disobedience	16 (5.9)	255 (17.4)	0.30	0.18–0.50	< 0.001
Prior substances use	118 (43.5)	410 (27.9)	1.99	1.52–2.59	< 0.001
Social withdrawal	25 (9.2)	222 (15.1)	0.57	0.37–0.88	0.010
Mood lability	19 (7.0)	143 (9.7)	0.70	0.42–1.15	0.155
Insomnia	117 (43.2)	274 (18.7)	3.31	2.51–4.35	< 0.001

*Note:* Data are presented per consultation.

Abbreviations: 95% CI = 95% confidence interval, OR = odds ratio.

Compared with their community‐living peers, detained adolescents were significantly more likely to have presented with insomnia, substance use, or suicide attempts (see also Table 4). Conversely, detained adolescents were less likely to present with irritability, agitation, aggressiveness, disobedience, attention difficulties, academic underachievement and social withdrawal. No significant differences were found for suicidal ideation, self‐mutilation, anxiety or psychotic symptoms (see also Table 4).

## 4 Discussion

Although psychiatric morbidity is frequently reported among detained adolescents, less is known about which clinical presentations are most closely associated with emergency psychiatric needs. Our findings suggest that acute stress‐related syndromes, suicidal behaviours, insomnia and substance‐related problems are the emergency presentations that most distinguish them from community‐living adolescents. Behavioural crises which might have been anticipated in the detained group, but were less likely as a clinical emergency need, suggest that the custodial services could often manage these, and that need for emergency clinical services was dictated by more traditionally clinical problems.

### 4.1 Sociodemographic Profile

The detained group consisted predominantly of boys and adolescents identifying as brown, with a mean age of 15.9 years, similar to reports from custodial youth populations in other countries (Bronsard et al. 2025; Wen et al. 2023; Teplin et al. 2021; Beaudry et al. 2021; Baglivio et al. 2017; Departamento Penitenciário Nacional 2017; Colins et al. 2010). However, compared with community‐living adolescents, detained adolescents were less likely to identify as White and more likely to identify as Black. In Brazil, black and brown adolescents aged 16 to 17 comprise a substantial proportion of the youth offender population, as reflected in data from the National System for Socio‐Educational Services (SINASE) (Departamento Penitenciário Nacional 2017).

The detained adolescents showed lower school attendance and were more likely to have repeated at least one school year, indicating substantial educational disruption. Together, these indicators likely reflect cumulative social adversity and developmental vulnerability, rather than a single explanatory factor.

### 4.2 Family History

Lower prevalence of psychiatric history in the families of the detained adolescents seems surprizing. It may reflect limitations in the available informants rather than true differences in family psychiatric history, and it is noted that many in this group were accompanied by institutional staff rather than relatives. Nevertheless, higher prevalence of family history of substance use disorder (SUD) was reported and aligns with previous findings (Acheson et al. 2018; Baglivio et al. 2017; Khoddam et al. 2015).

### 4.3 Diagnosis at Consultation

The strongest association with detained status was observed for stress reactions/adjustment disorders, conduct disorder, unspecified mood disorder and substance use disorders. Depression per se, although affecting a quarter of this group, was significantly less frequent than among the community‐living adolescents, whereas unspecified mood disorders were more frequent. This may indicate that mood presentations among detained adolescents were more difficult to characterise during emergency assessment; however, this hypothesis was not directly examined in the present study.

The slightly higher rate of substance use disorders among the detained group seems hardly to reflect international evidence of high substance‐related morbidity among young people under justice system detention (Teplin et al. 2021; Beaudry et al. 2021; Acheson et al. 2018; Ribas‐Siñol et al. 2015; Swendsen et al. 2010), but our study was of emergency presentations rather than a primary disorder prevalence study, and these figures probably reflect that. Similarly, the small difference between samples in intellectual disability diagnosis is probably more reflective of emergency needs than overall diagnostic differences (Fazel et al. 2008; Lunsky et al. 2008; Hayes 2007).

The most salient finding was the disproportionate association between detained status and stress‐related/adjustment disorders. Traumatic experiences are ubiquitous in this population and are likely to be the primary drivers of acute psychiatric destabilisation, perhaps exacerbated by the ‘institutional shock’ of confinement, reduced autonomy and environmental transition (Teplin et al. 2021; Baglivio et al. 2017; Godinet et al. 2014; Dória 2011).

### 4.4 Previous Diagnosis

Interpretation of prior psychiatric history requires caution due to missing data, which often reflects the fragmented clinical documentation within the justice system. Whereas international literature consistently reports elevated ADHD prevalence among justice‐involved youth (Abram et al. 2015; Beaudry et al. 2021), this pattern was not observed in the present sample but may simply reflect its lesser contribution to emergency psychiatric presentations—the focus of this study.

### 4.5 Reasons for Seeking Emergency Care

Prior substance use, insomnia, sadness, agitation, suicide attempts, aggressiveness and irritability were the most frequent presenting complaints among detained adolescents, reflecting a complex interplay between stress‐related affective symptoms and behavioural dysregulation. Compared with community‐living adolescents, detained adolescents had higher odds of suicide attempts and insomnia, despite similar suicidal ideation rates. This discrepancy may indicate greater impulsivity, reduced protective supports and heightened acute stress reactivity in restrictive environments (World Health Organization 2019; Beaudry et al. 2021; Dória 2011).

A critical distinction in this study is that substance‐related complaints were more likely to be related to the consequences of prior use rather than acute intoxication, as access to illicit substances is legally prohibited within custodial settings. Even in the absence of documented withdrawal states, substance‐related pathology may interact with sleep disturbance and stress dysregulation to amplify crisis risk (Teplin et al. 2021; Acheson et al. 2018; Baglivio et al. 2017; Ribas‐Siñol et al. 2015).

Insomnia, reported by nearly half of the detained adolescents, may have different implications for them than for community‐living adolescents. Rather than a primary mood disorder process, sleep disturbances in this context may reflect a combination of environmental transition, stress reactivity and substance‐related vulnerability (American Psychiatric Association 2022; Dória 2011). Also, because insomnia intensifies emotional instability, it may act as a catalyst for crisis escalation and increased suicide risk (American Psychiatric Association 2022; Costa and da Silva 2017; Dória 2011). Although agitation and aggressiveness were frequent among the detained adolescents, they were less so than among the community‐living group. This unexpected finding may suggest that many behavioural crises were successfully managed within custodial services, whereas referral to emergency psychiatric care was more often prompted by acute clinical conditions such as stress‐related symptoms, suicidality, sleep disturbance or substance‐related problems. Although this interpretation cannot be confirmed by the present study, it highlights an important distinction between behavioural management within custodial settings and indications for external emergency psychiatric assessment.

### 4.6 Limitations

This study has limitations inherent to its retrospective design and reliance on medical records. Clinical documentation was conducted under acute emergency circumstances, often based on brief interviews. For the detained adolescents, involvement of institutional staff rather than biological family as legal guardians likely limits some history detail. However, the inclusion of all eligible patients during the study period ensured a substantial sample size for key comparisons. In addition, as the study was conducted in a psychiatric emergency setting, the sample may overrepresent the most severe cases among detained adolescents. Custodial institutions may also have been better equipped than the wider community to manage some behavioural crises, even in the absence of on‐site psychiatrists, thereby influencing referral patterns. Consequently, these findings may not be generalisable to the broader justice‐involved population, such as those under community‐based supervision. Finally, while standardized screening instruments were not used, all diagnoses were established according to ICD‐10 criteria by board‐certified child and adolescent psychiatrists. This approach reflects routine clinical practice in public health systems, thereby supporting the clinical validity of our findings.

Despite these limitations, the study provides valuable real‐world evidence from a large public psychiatric emergency service, providing important insights into the acute mental health needs of detained adolescents.

## 5 Conclusions

Detained adolescents represent a highly vulnerable group who may have psychiatric emergency needs. Our findings suggest that, although conduct disorder was more common among detained adolescents, emergency psychiatric presentations were more strongly characterised by stress‐related disorders, suicide attempts and insomnia. This suggests that psychiatric destabilisation in custodial settings is driven primarily by affective and acute stress responses rather than chronic behavioural patterns alone.

These findings are compatible with the increasingly recognised need for trauma‐informed care and stress‐regulation interventions within the juvenile justice system. They also highlight the importance of emergency psychiatric assessment extending beyond behavioural disturbances to include careful evaluation of stress‐related disorders, suicidal behaviour, sleep disturbances and substance‐related problems. Whether interventions specifically targeting these clinical needs improve outcomes remains an important question for future research.

## Author Contributions

**Maria Carolina Lobato Machado:** conceptualization, data curation, formal analysis, investigation, methodology, project administration, resources, supervision, writing – original draft, writing – review and editing. **Marcela de Moura Rezende:** investigation, writing – original draft. **Izabela Lara Borém:** investigation, writing – original draft. **Maria Eugênia Rezeck Braga Hibner:** investigation. **Danilo Barcellos Nogueira:** investigation. **Luciana Rodrigues da Cunha:** investigation, resources, supervision. **Rodrigo Nicolato:** supervision, writing – review and editing. **Debora Marques de Miranda:** methodology, supervision, writing – review and editing. All authors have read and approved the final version of the manuscript.

## Funding

One of the authors received support through the institutional scholarship programme of Faculdade Ciências Médicas de Minas Gerais. The scholarship provider had no role in the design or conduct of the study; data collection, analysis or interpretation; preparation, review or approval of the manuscript or the decision to submit the manuscript for publication. No specific funding was received for this research.

## Ethics Statement

This study was approved by the Research Ethics Committee (CAAE: 83419717.0.0000.5134; approval date: 25 October 2017). As this was a retrospective chart review with no direct contact with patients or their families, informed consent and assent were waived by the Committee. All procedures followed ethical principles for research with human subjects, ensuring confidentiality and data protection. Date of birth and medical record number were used solely for record linkage before anonymisation.

## Consent

Patient consent was waived by the Research Ethics Committee because this was a retrospective chart review using anonymised medical records.

## Conflicts of Interest

The authors declare no conflicts of interest.

## Data Availability

The data that support the findings of this study are openly available in the Open Science Framework (OSF) at https://osf.io/7x65y, with the following DOI: https://doi.org/10.17605/OSF.IO/SK67R. The dataset has been fully anonymised to protect participant confidentiality and is shared in accordance with ethical guidelines. These data are freely accessible and comply with the FAIR principles.

## References

[cbm70051-bib-0001] Abram, K. M. , L. A. Teplin , G. M. McClelland , and M. K. Dulcan . 2003. “Comorbid Psychiatric Disorders in Youth in Juvenile Detention.” Archives of General Psychiatry 60, no. 11: 1097–1108. 10.1001/archpsyc.60.11.1097. PMC2893728 14609885

[cbm70051-bib-0002] Abram, K. M. , N. A. Zwecker , L. J. Welty , J. A. Hershfield , M. K. Dulcan , and L. A. Teplin . 2015. “Comorbidity and Continuity of Psychiatric Disorders in Youth After Detention: A Prospective Longitudinal Study.” JAMA Psychiatry 72, no. 1: 84–93. 10.1001/jamapsychiatry.2014.1375. PMC4562696 25426584

[cbm70051-bib-0003] Acheson, A. , A. S. Vincent , A. J. Cohoon , and W. R. Lovallo . 2018. “Defining the Phenotype of Young Adults With Family Histories of Alcohol and Other Substance Use Disorders: Studies From the Family Health Patterns Project.” Addictive Behaviors 77: 247–254. 10.1016/j.addbeh.2017.10.014. PMC6741351 29107202

[cbm70051-bib-0004] American Psychiatric Association . 2022. Diagnostic and Statistical Manual of Mental Disorders: DSM‐5‐TR. 5th ed., text rev. American Psychiatric Association Publishing.

[cbm70051-bib-0005] Baglivio, M. T. , K. T. Wolff , A. R. Piquero , M. A. Greenwald , and N. Epps . 2017. “Racial/Ethnic Disproportionality in Psychiatric Diagnoses and Treatment in a Sample of Serious Juvenile Offenders.” Journal of Youth and Adolescence 46, no. 7: 1424–1451. 10.1007/s10964-016-0573-4. 27665279

[cbm70051-bib-0006] Beaudry, G. , R. Yu , N. Långström , and S. Fazel . 2021. “An Updated Systematic Review and Meta‐Regression Analysis: Mental Disorders Among Adolescents in Juvenile Detention and Correctional Facilities.” Journal of the American Academy of Child & Adolescent Psychiatry 60, no. 1: 46–60. 10.1016/j.jaac.2020.01.015. PMC8222965 32035113

[cbm70051-bib-0007] Brasil . 1990. Lei n° 8.069, de 13 de Julho de 1990: Estatuto da Criança e do Adolescente. Diário Oficial da União. http://www.planalto.gov.br/ccivil_03/leis/l8069.htm.

[cbm70051-bib-0008] Brasil, Conselho Nacional de Justiça . 2012. Panorama Nacional: A Execução de Medida Socioeducativa de Internação. https://www.cnj.jus.br/wp‐content/uploads/2011/02/panorama_nacional_doj_web.pdf.

[cbm70051-bib-0009] Bronsard, G. , P. A. Leroux , I. Diallo , et al. 2025. “Prevalence of Mental Disorders in Closed Educational Centers in France.” Encephale 51, no. 1: 15–21. 10.1016/j.encep.2023.11.026. 38368185

[cbm70051-bib-0010] Colins, O. , R. Vermeiren , C. Vreugdenhil , W. van den Brink , T. Doreleijers , and E. Broekaert . 2010. “Psychiatric Disorders in Detained Male Adolescents: A Systematic Literature Review.” Canadian Journal of Psychiatry 55, no. 4: 255–263. 10.1177/070674371005500409. 20416149

[cbm70051-bib-0011] Costa, N. R. , and P. R. F. da Silva . 2017. “A Atenção em Saúde Mental Aos Adolescentes em Conflito Com a Lei no Brasil.” Ciência & Saúde Coletiva 22, no. 5: 1467–1478. 10.1590/1413-81232017225.33562016. 28538918

[cbm70051-bib-0014] Departamento Penitenciário Nacional . 2017. “Levantamento Nacional de Informações Penitenciárias—Infopen.” http://antigo.depen.gov.br/DEPEN/depen/sisdepen/infopen.

[cbm70051-bib-0015] Dória, G. M. 2011. “Avaliação Dos Transtornos Psiquiátricos em Adolescentes em Conflito Com a Lei.” Doctoral dissertation, Universidade Federal do Paraná. https://acervodigital.ufpr.br/handle/1884/36834.

[cbm70051-bib-0016] Fazel, S. , K. Xenitidis , and J. Powell . 2008. “The Prevalence of Intellectual Disabilities Among 12,000 Prisoners: A Systematic Review.” International Journal of Law and Psychiatry 31, no. 4: 369–373. 10.1016/j.ijlp.2008.06.001. 18644624

[cbm70051-bib-0017] Gama, C. A. P. , R. T. O. Campos , and A. L. Ferrer . 2014. “Saúde Mental e Vulnerabilidade Social: A Direção do Tratamento.” Revista Latinoamericana de Psicopatologia Fundamental 17, no. 1: 69–84. 10.1590/s1415-47142014000100006.

[cbm70051-bib-0018] Godinet, M. T. , F. Li , and T. Berg . 2014. “Early Childhood Maltreatment and Trajectories of Behavioral Problems: Exploring Gender and Racial Differences.” Child Abuse & Neglect 38, no. 3: 544–556. 10.1016/j.chiabu.2013.07.018. 23993147

[cbm70051-bib-0019] Hayes, S. C. 2007. “People With Intellectual Disabilities and the Criminal Justice System: Two Rural Courts.” Journal of Intellectual Disability Research 51, no. 7: 525–536. 10.1111/j.1365-2788.2006.00884.x.

[cbm70051-bib-0020] Khoddam, R. , M. Worley , K. C. Browne , N. Doran , and S. A. Brown . 2015. “Family History Density Predicts Long‐Term Substance Use Outcomes in an Adolescent Treatment Sample.” Drug and Alcohol Dependence 147: 235–242. 10.1016/j.drugalcdep.2014.11.009. PMC4297729 25533896

[cbm70051-bib-0021] Lim, Y. , E. J. Park , and B. Kim . 2018. “Psychiatric Disorders and Recidivism Among Korean Adolescents on Probation or Parole.” Psychiatry Investigation 15, no. 6: 561–567. 10.30773/pi.2017.11.30.1. PMC6018139 29788699

[cbm70051-bib-0022] Lo, C. B. , J. A. Bridge , J. Shi , L. Ludwig , and R. M. Stanley . 2020. “Children’s Mental Health Emergency Department Visits: 2007–2016.” Pediatrics 145, no. 6: e20191536. 10.1542/peds.2019-1536. 32393605

[cbm70051-bib-0023] Lunsky, Y. , C. Gracey , and S. Gelfand . 2008. “Emergency Psychiatric Services for Individuals With Intellectual Disabilities: Perspectives of Hospital Staff.” Intellectual and Developmental Disabilities 46, no. 6: 446–455. 10.1352/2008.46:446-455. 19006431

[cbm70051-bib-0024] Mutlu, C. , A. G. Kilicoglu , H. Gunes , et al. 2015. “Characteristics of Pediatric Psychiatric Emergency Population in a Mental Health Hospital.” Eurasian Journal of Emergency Medicine 14, no. 3: 123–130. 10.5152/eajem.2015.71354.

[cbm70051-bib-0025] Porter, M. , R. Gracia , J. C. Oliva , M. Pàmias , G. Garcia‐Parés , and J. Cobo . 2016. “Mental Health Emergencies in Paediatric Services: Characteristics, Diagnostic Stability, and Gender Differences.” Actas Españolas de Psiquiatría 44, no. 6: 203–211. PMID: 27906411. 27906411

[cbm70051-bib-0026] Resende, A. Z. , and F. M. Volpe . 2021. “A Disparidade Entre a Demanda e a Oferta de Psiquiatras da Infância e Adolescência em Minas Gerais.” RAHIS‐Revista de Administração Hospitalar e Inovação em Saúde 18, no. 4: 1–13. 10.21450/rahis.v18i4.7286.

[cbm70051-bib-0027] Ribas‐Siñol, M. , N. Del Prado‐Sanchez , J. Claramunt‐Mendoza , M. Civit‐Ramirez , O. Canalias‐Perez , and S. Ochoa . 2015. “Troubled Adolescents: Substance Abuse and Mental Disorder in Young Offenders.” Actas Españolas de Psiquiatría 43, no. 6: 197–204. PMID: 26631302. 26631302

[cbm70051-bib-0028] Swendsen, J. , K. P. Conway , L. Degenhardt , et al. 2010. “Mental Disorders as Risk Factors for Substance Use, Abuse and Dependence: Results From the 10‐Year Follow‐Up of the National Comorbidity Survey.” Addiction 105, no. 6: 1117–1128. 10.1111/j.1360-0443.2010.02902.x. PMC2910819 20331554

[cbm70051-bib-0029] Teplin, L. A. , L. M. Potthoff , D. A. Aaby , L. J. Welty , M. K. Dulcan , and K. M. Abram . 2021. “Prevalence, Comorbidity, and Continuity of Psychiatric Disorders in a 15‐Year Longitudinal Study of Youths Involved in the Juvenile Justice System.” JAMA Pediatrics 175, no. 7: e205807. 10.1001/jamapediatrics.2020.5807. PMC8022269 33818599

[cbm70051-bib-0030] Wen, A. , N. R. Gubner , M. M. Garrison , and S. C. Walker . 2023. “Racial Disparities in Youth Pretrial Detention: A Retrospective Cohort Study Grounded in Critical Race Theory.” Health & Justice 11, no. 1: 14. 10.1186/s40352-022-00203-8. PMC9993616 36882535

[cbm70051-bib-0032] World Health Organization . 2019. “Suicide in the World: Global Health Estimates.” https://apps.who.int/iris/handle/10665/326948.

